# Roles of Pyruvate, NADH, and Mitochondrial Complex I in Redox Balance and Imbalance in *β* Cell Function and Dysfunction

**DOI:** 10.1155/2015/512618

**Published:** 2015-10-19

**Authors:** Xiaoting Luo, Rongrong Li, Liang-Jun Yan

**Affiliations:** ^1^Department of Pharmaceutical Sciences, UNT System College of Pharmacy, University of North Texas Health Science Center, 3500 Camp Bowie Boulevard, Fort Worth, TX 76107, USA; ^2^Department of Biochemistry and Molecular Biology, Gannan Medical University, Ganzhou, Jiangxi 341000, China

## Abstract

Pancreatic *β* cells not only use glucose as an energy source, but also sense blood glucose levels for insulin secretion. While pyruvate and NADH metabolic pathways are known to be involved in regulating insulin secretion in response to glucose stimulation, the roles of many other components along the metabolic pathways remain poorly understood. Such is the case for mitochondrial complex I (NADH/ubiquinone oxidoreductase). It is known that normal complex I function is absolutely required for episodic insulin secretion after a meal, but the role of complex I in *β* cells in the diabetic pancreas remains to be investigated. In this paper, we review the roles of pyruvate, NADH, and complex I in insulin secretion and hypothesize that complex I plays a crucial role in the pathogenesis of *β* cell dysfunction in the diabetic pancreas. This hypothesis is based on the establishment that chronic hyperglycemia overloads complex I with NADH leading to enhanced complex I production of reactive oxygen species. As nearly all metabolic pathways are impaired in diabetes, understanding how complex I in the *β* cells copes with elevated levels of NADH in the diabetic pancreas may provide potential therapeutic strategies for diabetes.

## 1. Introduction

**Figure 1:**
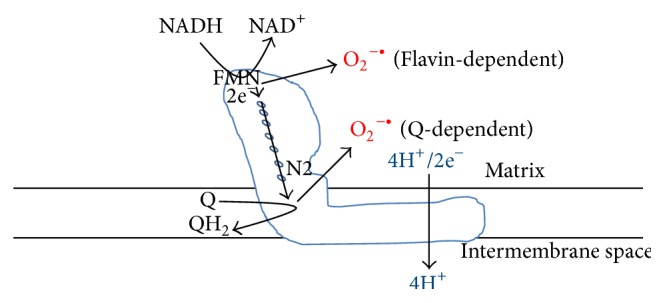
The three key roles of mitochondrial complex I: NADH oxidation and recycling, superoxide production, and proton pumping. Electrons from NADH are transported to CoQ via seven Fe-S clusters with the terminal one being N2 [6]. Superoxide could be produced at both the FMN-dependent site and the CoQ-dependent site [132].

Complex I (NADH-ubiquinone oxidoreductase) is the primary electron entry point in mitochondrial electron transport chain [1, 2] and is absolutely required for glucose-stimulated insulin secretion [3]. In mammalian cells, complex I has at least 45 subunits with a molecular weight close to 1000 kDa [4–6]. This huge complex has three major functions in mitochondrial bioenergetics and oxygen consumption (Figure 1). First, it is the major enzyme that oxidizes NADH to NAD^+^; thus, it is responsible for regenerating the majority of NAD^+^ for continued glycolysis and for the function of NAD^+^-dependent enzymes such as sirtuins, CD38, and poly ADP ribose polymerases (PARPs) [7–11]. Second, complex I is the major proton pumping machine in the mitochondrial inner membrane [2, 12], which drives mitochondrial ATP production needed by nearly all cells. Third, complex I is the major site for cellular production of reactive oxygen species (ROS) [13, 14] that have been demonstrated to be involved in cell survival and death mechanisms [15, 16]. Interestingly, despite numerous studies on complex I and its implications in a variety of diseases [17–22], the role of complex I in *β* cells in the diabetic pancreas remains unknown, albeit normal function of complex I [3] and a basal level of complex I-generated ROS are needed for insulin secretion under physiological conditions [23]. In this review, we discuss the fate of glucose, mechanisms of insulin secretion, and the roles of glucose metabolic pathways including pyruvate cycling and NADH cycling in insulin secretion under physiological conditions. We propose the hypothesis that complex I is a key player in maintaining redox balance for *β* cell insulin secretion and that its dysfunction impairs *β* cell function.

## 2. Fate of Glucose under Physiological Conditions

**Figure 2:**
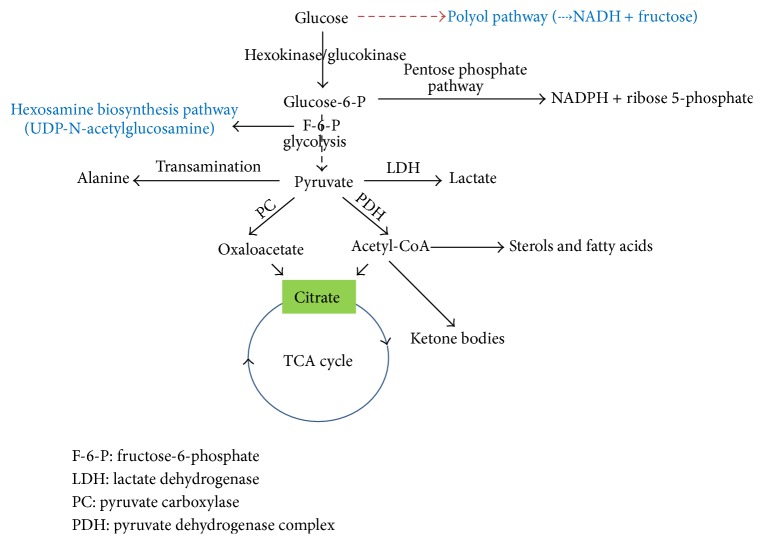
Fate of glucose. The major pathways shown in this diagram are glycolysis, the TCA cycle, and the pentose phosphate pathway. Additionally, glucose can be used as sources for fatty acid synthesis and lactate and alanine formation and can also be stored as glycogen in liver and skeletal muscle (not shown in the diagram). The pathways in blue (the polyol pathway and the hexosamine pathway) can be significant ones for glucose utilization under diabetic conditions.

Glucose can be metabolized to form numerous biomolecules [24, 25] (Figure 2). It is used for ATP synthesis via the glycolytic and oxidative phosphorylation pathways. It generates the reducing equivalent NADPH for anabolism and ribose 5-phosphate for nucleotide synthesis via the pentose phosphate pathway [26]. It can be converted via pyruvate to alanine and can also be converted to lactate when the oxygen supply is limited [24]. Furthermore, ketone bodies, sterols, and fatty acids can all be synthesized from glucose via pyruvate-derived acetyl-CoA [24].

## 3. *β* Cell Glucose Sensing and Insulin Secretion

**Figure 3:**
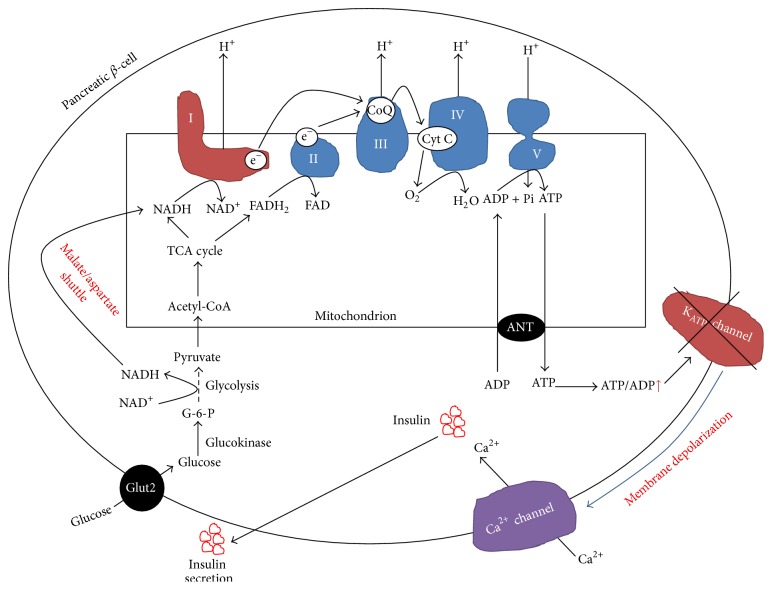
Mechanisms of *β* cell glucose sensing and insulin secretion. Shown is the first phase of insulin secretion stimulated by glucose derived ATP. When glucose levels are high, ATP levels are high, which depolarizes cell membranes, triggers the closure of the K_ATP_ channels, and induces opening of the Ca^2+^ channel. Consequently, insulin granules are infused with membranes and insulin is released. Complex I plays a key role in this process as ATP production is driven by its oxidation of NADH and transportation of electrons to CoQ that accompany proton pumping needed for ATP synthesis by complex V.

In *β* cells, glucose not only is a fuel, but also stimulates insulin secretion [27–30]. Because *β* cells have a high-Km glucose transporter 2 (Glut2) and high-Km glucokinase, they can respond to elevated levels of blood glucose, which is known as supply-driven metabolism [31, 32]. Therefore, there is a tight link between glucose metabolism and insulin secretion [33–35]. The canonical events that trigger insulin secretion after a meal are outlined in Figure 3 [35–38]. Glucose is transported into *β* cells by the glucose transporter 2 (Glut2). Once inside the cells, glucose is phosphorylated by glucokinase to yield glucose-6-phosphate (G-6-P) [39, 40], which is then converted to 2 molecules of pyruvate by the glycolytic pathway. Pyruvate is then transported into mitochondria and converted to acetyl-CoA by the pyruvate-dehydrogenase complex. Acetyl-CoA then enters the tricarboxylic acid (TCA) cycle and electrons derived from it are donated to NAD^+^ and FAD, leading to generation of intramitochondrial NADH and FADH_2_. Electrons stored in these two molecules are further donated to coenzyme Q (CoQ) via complex I and complex II, respectively. The eventual electron transportation to O_2_ leads to a proton gradient formation across the inner mitochondrial membranes, which drives ATP synthesis via complex V. When blood glucose levels are elevated, more NADH and ATP are produced, leading to closure of ATP-sensitive potassium channels, which in turn depolarizes cell membranes and consequently opens voltage-gated Ca^2+^ channels, resulting in Ca^2+^ influx into the cells [30]. It is this Ca^2+^ influx that triggers the initial phase of insulin secretion from prestored insulin granules after nutrient ingestion (Figure 3) [28, 35, 41].

Once stored insulin is depleted, a second phase of insulin release is initiated [42, 43]. This phase of insulin release is K_ATP_ channel-independent [29] and this phase is prolonged as insulin has to be synthesized, processed, and released for the length of time of elevated blood glucose. This phase also regenerates stores of insulin depleted in the first phase of insulin secretion and is likely stimulated by metabolites such as NADPH and *α*-ketoglutarate produced by pyruvate cycling pathways involving TCA cycle intermediates such as citrate, malate, and oxaloacetate [29, 37, 44].

## 4. Pyruvate Cycling, Conversion of NADH to NADPH, and Insulin Secretion

**Figure 4:**
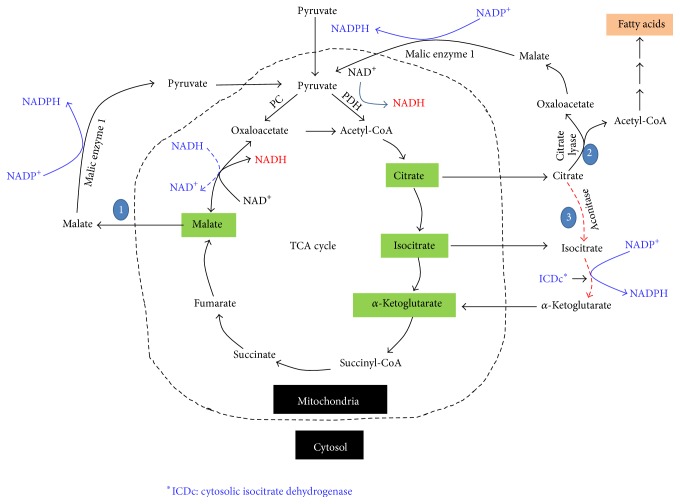
The pyruvate recycling pathways in *β* cell mitochondria. The three pathways shown are pyruvate-malate pathway, the pyruvate-citrate pathway, and the pyruvate-isocitrate pathway. Each pathway converts reducing equivalents from NADH to NADPH that plays key roles in the second phase of insulin secretion.

As an intermediate of glucose metabolism in *β* cells, pyruvate plays an important role in redox cycling between NADH and NADPH [41, 45, 46]. This is reflected by the three pyruvate cycling pathways across the mitochondrial membranes (Figure 4). The first is pyruvate-malate pathway. In this pathway, pyruvate is converted to oxaloacetate by pyruvate carboxylase. The latter is converted to malate by mitochondrial malate dehydrogenase. Malate is then shuttled out of mitochondria to the cytosol whereby it is converted back to pyruvate. This process results in the net formation of NADPH from NADH. The second pathway is the pyruvate-citrate pathway, in which citrate is transported out of mitochondria into the cytosol whereby it is split by citrate lyase to yield acetyl-CoA and oxaloacetate. Acetyl-CoA can be used as the carbon source for fatty acid synthesis and oxaloacetate can be converted by malic enzyme 1 to pyruvate that then reenters mitochondria. Similar to the pyruvate-malate pathway, the pyruvate-citrate pathway also results in the net formation of NADPH from NADH. The third pathway is pyruvate-isocitrate pathway involving cytosolic isocitrate dehydrogenase that uses NADP as its cofactor [47]. Therefore, reducing equivalents again are transferred from NADH to NADPH.

Evidence supporting the role of the three pyruvate cycling pathways and NADPH in insulin secretion comes mainly from the following studies. (A) Both pharmacological inhibitors and siRNA-mediated suppression of mitochondrial pyruvate carrier severely impair insulin secretion [48]. (B) siRNA-mediated suppression of malic enzyme 1 impairs insulin secretion [49]. (C) *β* cells have high levels of pyruvate carboxylase activity [44, 50]. Unlike liver and kidney cells that have phosphoenolpyruvate carboxykinase (PEPCK) used for gluconeogenesis, *β* cells do not have detectable PEPCK [44, 51]. Therefore, *β* cell pyruvate carboxylase must have a purpose other than gluconeogenesis, which is thought to replenish oxaloacetate in the TCA cycle when oxaloacetate is removed for the pyruvate-malate pathway to generate NADPH [52]. It has been reported that in *β* cells nearly 50% of the pyruvate pool derived from glucose is converted to oxaloacetate [53]. Oxaloacetate not only replenishes the TCA cycle intermediates, but also drives the pyruvate-malate cycling pathway for NADPH production. Therefore, pyruvate and NADPH are thought to be essential triggers for *β* cell insulin secretion [54–57].

## 5. Production and Recycling of NADH

### 5.1. NADH Production Pathways

**Figure 5:**
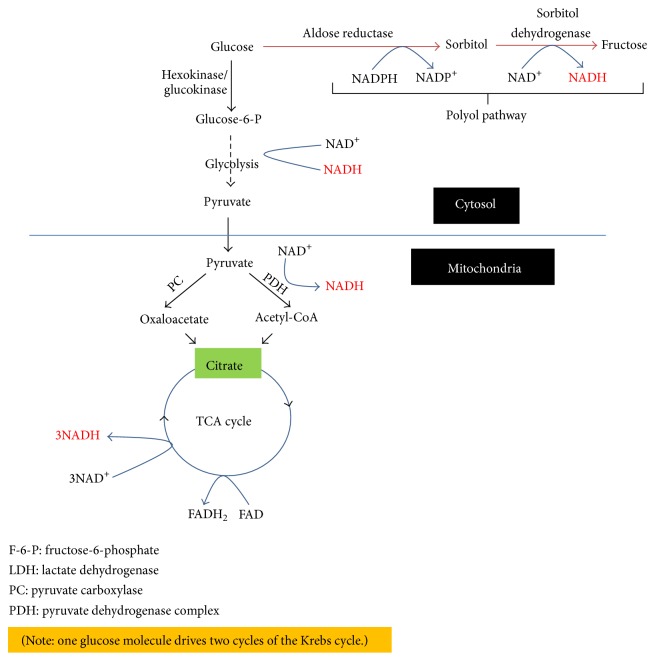
Pathways of NADH production during glucose combustion. These are the glycolytic pathway, the pyruvate dehydrogenase complex, and the TCA cycle. Under diabetic conditions, the polyol pathway also becomes a significant pathway for NADH production that can further perturb the redox balance between NADH and NAD^+^.

Electrons derived from glucose metabolism are stored in NADH and FADH_2_, respectively. In terms of glucose combustion, NADH is mainly generated by the glycolytic pathway, by pyruvate dehydrogenase complex via dihydrolipoamide dehydrogenase [58], and by the TCA cycle [59]. As shown in Figure 5, degradation of one molecule of glucose can yield 8 molecules of NADH and two molecules of FADH_2_ (note that one molecule of glucose drives two cycles of the TCA cycle).

### 5.2. Redox Shuttles for NADH Transportation

**Figure 6:**
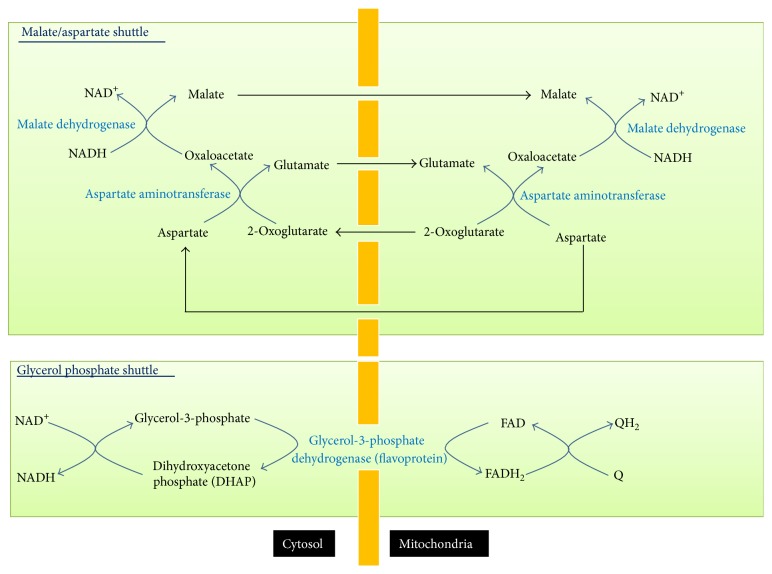
The two redox shuttles transporting cytosolic NADH into mitochondria. These are malate/aspartate shuttle and the glycerol phosphate shuttle. The former is reversible and only transports NADH when cytosolic NADH levels are high; the latter is irreversible and can transport NADH from cytosol to mitochondria even when cytosolic NADH levels are low.

For *β* cells, NADH produced during glycolysis is required for glucose sensing [60] and has to be transported into mitochondria for oxidation by complex I. This is because, while in most tissues lactate dehydrogenase can regenerate NAD^+^ for glycolysis to continue, *β* cells have very low lactate dehydrogenase activity [61]. The translocation of NADH from cytoplasm to mitochondria is achieved by two redox shuttles (Figure 6): the malate-aspartate shuttle and the glycerol phosphate shuttle [62–64]. While the malate-aspartate shuttle can directly feed NADH to complex I, the glycerol phosphate shuttle only transports electrons from NADH to FADH_2_ that donates its electrons to CoQ via complex II. Therefore, the glycerol phosphate shuttle is less efficient in terms of energy production [24]. Another difference between the two redox shuttles is that the malate-aspartate shuttle is a reversible process that can only be activated by high level cytosolic NADH, while the glycerol phosphate shuttle is an irreversible process that can transport NADH into mitochondria even when the cytosolic NADH level is low [24]. The two redox shuttles have been shown to be important for glucose-induced insulin secretion [63–65] as blocking of both shuttles abolished glucose-induced insulin secretion although deficiency of either shuttle singly did not alter the response to glucose stimulation [28].

### 5.3. Complex I and NADH Recycling

Under aerobic oxidation, nearly all NADH molecules generated by glycolysis in *β* cells will need to be recycled by complex I (Figure 1) so that further glucose degradation can continue. It is known that even under resting conditions the rates of NADH and pyruvate generation are faster than the rates they are used [66]. Therefore, we propose that complex I is the major enzyme maintaining NAD^+^/NADH redox balance and should be under constant electron pressure. While complex I's oxidation of NADH is the major pathway for NADH/NAD^+^ recycling, part of NADH can also be used for reducing equivalent transfer to NADPH via the pyruvate cycling pathways as shown in Figure 4. However, whether there is any crosstalk between complex I and the pyruvate cycling pathways remains unknown at the present time.

## 6. Fate of Glucose in Diabetes

Under diabetic conditions, the glycolytic pathway is usually impaired, not only due to inhibition by elevated levels of NADH resulting from overnutrition or fuel excess [25, 67], but also due to impairment of glycerol-3-phosphate dehydrogenase that is very vulnerable to oxidative and posttranslational modifications [68–71]. The consequence of this impairment is that the flux of glucose through otherwise insignificant glucose metabolic pathways is increased. These include the polyol pathway and the hexosamine pathway (Figure 2, the pathways in blue), PKC activation, and the advanced glycation pathway [72]. Each of these pathways has been demonstrated to be involved in ROS production and induction of oxidative stress [71]. Therefore, oxidative stress has been postulated to be a unifying mechanism by which diabetes and its complications develop [73, 74].

## 7. The Polyol Pathway and NADH/NAD^+^ Redox Imbalance

**Figure 7:**
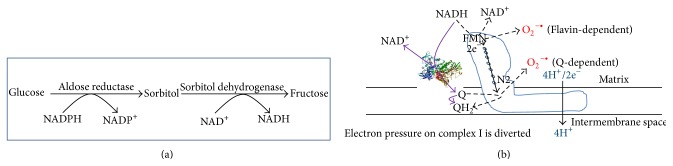
(a) The polyol pathway becomes a major pathway for NADH production under diabetic conditions. NADH is produced in the second reaction catalyzed by sorbitol dehydrogenase. (b) Proposed approach that may alleviate complex I NADH pressure and minimize superoxide production. This could be achieved by a molecule (a protein or a chemical) that can transport electrons from NADH directly to CoQ by bypassing complex I. As this bypass electron transport occurs without proton pumping, no superoxide should be produced, thereby facilitating NADH oxidation and minimizing superoxide production.

Since the polyol pathway generates NADH that can be fed into complex I via the malate-aspartate shuttle, we would like to discuss the role of this pathway in diabetes in a little more detail. The pathway involves two steps (Figure 7(a)). The first reaction is glucose reduction by aldose reductase to form sorbitol. This step consumes NADPH, so NADP^+^ is formed. In certain tissues, sorbitol can accumulate and impair cellular function by altering osmolarity [75, 76]. The second reaction is sorbitol oxidation by sorbitol dehydrogenase to form fructose. This reaction uses NAD^+^ as the oxidant and generates NADH and has been thought to be a major contributing factor to NADH/NAD^+^ redox imbalance and pseudohypoxia as it can compete with GAPDH for NAD^+^ [77], thereby decreasing cytosolic level of NAD^+^ [78–80]. Intriguingly, the rates of both glycolysis and the polyol pathway are known to be increased in diabetic hyperglycemia [76], but how complex I handles the additional amount of NADH produced by the polyol pathway is unknown. Moreover, it should be noted that the accumulation of fructose has been suggested to be more deleterious than that of glucose [75, 81] as fructose metabolism by fructokinase bypasses key-regulated steps of the glycolytic pathway [82] and thus can deplete intracellular phosphate and ATP, thereby inducing oxidative stress and inflammation [83].

Interestingly, as the first reaction consumes NADPH, it has been suggested that consumption of NADPH by the polyol pathway can also contribute to oxidative stress because a lower level of NADPH would impair glutathione synthesis by NADPH-dependent glutathione reductase. However, conclusive evidence that NADPH levels or alterations in NADPH/NADP^+^ ratios are lower in diabetes has yet to be established. In fact, it has been reported that NADPH levels in certain diabetic tissues are higher [75, 76, 84], though the underlying mechanisms remain unknown. It is likely that the pyruvate cycling pathways could generate the majority of NADPH in diabetes.

## 8. Complex I and *β* Cell Dysfunction in the Diabetic Pancreas

During diabetes, many metabolic pathways are impaired due to persistent hyperglycemia. At the early stages of hyperglycemia, elevated levels of NADH are mainly produced by the conventional glucose metabolic pathways including glycolysis and the TCA cycle. As more NADH is produced, more electron pressure would be imposed on complex I. In this sense, complex I dysfunction would likely mean increased complex I activities as more NADH needs to be handled by complex I. Indeed, it has been reported that complex I activity is elevated in streptozotocin-induced diabetic rats [85, 86]. Furthermore, as NADH oxidation by complex I is accompanied by electron flow associated with electron leakage and partial oxygen reduction [87, 88], more NADH oxidation would thus lead to more ROS production [89]. This would eventually impair the glycolytic pathway due to inhibition of glycerol-3-phosphate dehydrogenase by reduced availability of NAD^+^ [68, 69, 71, 90–94], leading to diversion of glucose to other disposal pathways such as the polyol pathway [95, 96]. It has been estimated that under diabetes approximately 30% of the glucose is metabolized by the polyol pathway [93]. As this pathway generates NADH from NAD^+^, the ratio of NADH to NAD^+^ is highly elevated and perturbed [73, 79, 97], leading to enhanced ROS production [98, 99] and establishment of a chronic pseudohypoxic condition that can cause chronic inflammation known to be contributing to *β* cell dysfunction [100–102]. Hence, there is a problem in NADH and NAD^+^ recycling in diabetes, suggesting that complex I function is impaired. We incline that complex I activity would be elevated in diabetic pancreas as more NADH has to be recycled by complex I. Nonetheless, how complex I function is indeed impaired (either an increase or a decrease in activity) by diabetic hyperglycemia in *β* cells has yet to be investigated. It is our belief that, under diabetic conditions, a smooth flow of NADH via complex I could help fight diabetes. On one hand, NADH is overproduced due to overnutrition and hyperglycemic activation of the polyol pathway [81, 103]. On the other hand, the NAD^+^ level is getting lower and possibly facing depletion due to potential impairment in complex I activity and activation of NAD^+^-dependent enzymes such as sirtuins, CD38, and poly ADP ribose polymerase [10, 104–106]. Indeed, it has been established that overactivation of the NAD^+^-dependent PARP can trigger cell death due to NAD^+^ depletion [69, 107, 108]. Therefore, an efficient NADH oxidation by complex I in diabetes would be beneficial for diabetic individuals.

Based on the above discussions, we postulate that complex I represents a potential therapeutic target for diabetes. Specifically, as proposed in a hypothetical model shown in Figure 7(b), if a protein or a small molecule target could be designed under diabetic conditions to reduce metabolic pressure on complex I, that is, relaying excess electrons from NADH to CoQ, such a target could serve as a potential therapeutic approach by restoring NADH/NAD^+^ redox balance in the absence of enhanced proton pumping and ROS production. Future studies should be directed toward exploring these strategies.

Finally, it should be pointed out that while ROS have been thought to be involved in impairment of *β* cell function and insulin secretion, no clear evidence that antioxidants lower blood glucose in clinical settings has been reported. Nonetheless, in animal models of diabetes induced by streptozotocin, many compounds, particularly those from plants and herbs, have been shown to be able to lower blood glucose by scavenging ROS and attenuating oxidative stress [109–118]. The hypoglycemic effects of these compounds in human diabetes, however, remain to be fully evaluated. Additionally, it should also be pointed out that while both metformin and berberine have been shown to lower blood glucose levels by inhibiting complex I function [119–125], how they exert their actions on *β* cell complex I also remains to be investigated.

## 9. Summary

In this paper, we have summarized the glucose metabolic pathways and the roles of metabolic intermediates pyruvate and NADH in *β* cell function and insulin secretion. While the role of pyruvate recycling has been well established in *β* cell insulin secretion, the roles of NADH and complex I are yet to be fully elucidated. We thus focus our perspectives in this review on mitochondrial complex I that may contribute to redox balance under normal conditions and imbalance in diabetic conditions. We point out the fact that while complex I regulates NADH/NAD^+^ recycling [126] and ROS production under physiological conditions [127], its role in diabetes whereby redox balance between NADH and NAD^+^ is perturbed remains unexplored. We indicate that NADH overproduction due to chronic hyperglycemia would overload complex I, causing elevated levels of ROS production that has been previously postulated to contribute to the impairment of *β* cell function and insulin secretion [128–131]. Finally, we propose a hypothetic model of correcting this complex I-associated problem by alleviating complex I electron pressure that would also diminish complex I ROS production (Figure 7(b)). Future testing of this hypothesis may provide a potential therapeutic strategy for diabetes.
